# Short-Term Outcomes Following Cemented Versus Cementless Robotic-Assisted Total Knee Arthroplasty

**DOI:** 10.7759/cureus.30667

**Published:** 2022-10-25

**Authors:** Brian P McCormick, Paolo Rigor, Sarah M Trent, Ji Won Lee, Eshetu Tefera, Katherine L Mistretta, Henry R Boucher

**Affiliations:** 1 Orthopedics, MedStar Union Memorial Hospital, Baltimore, USA; 2 Orthopedics, Georgetown University School of Medicine, Washington, USA; 3 Research, Medstar Health Research Institute, Hyattsville, USA

**Keywords:** cemented, pressfit, robotics, total knee arthroplasty, implant fixation

## Abstract

Background: Cemented fixation during total knee arthroplasty (TKA) has long been the gold standard due to excellent survivorship and clinical outcomes. With recent biomaterial advancements, cementless fixation has gained renewed interest. Most studies demonstrate similar clinical outcomes and survivorship between these two fixation methods, without consensus regarding the optimal method of fixation during TKA. Outcomes following TKA also depend upon the proper alignment and positioning of components. Robotic-assisted TKA has been shown to improve outcomes related to component positioning, overall lower limb alignment, and soft tissue balancing. No study to date has investigated the role of robotic-assisted surgery on postoperative outcomes following cementless versus cemented TKA.

Methods: This is a retrospective cohort study of patients 18 years of age and older who underwent primary robotic-assisted TKA performed by a single fellowship-trained arthroplasty surgeon. Oxford Knee Scores and Short Form Health Survey scores were obtained preoperatively and at a two-year follow-up. Complications such as DVT, infection, arthrofibrosis requiring manipulation, and revision surgery were collected.

Results: Three hundred eighty knees in the cementless cohort and 72 cemented knees were included for analysis. There were no statistically significant differences between the two cohorts in terms of SF-12, Oxford Knee Scores, complications, or revision surgery rates.

Conclusion: Cementless fixation during TKA offers an alternative to cemented fixation with similar short-term results in terms of patient-reported outcomes, complication rates, and revision surgery rates. Further research is warranted to better understand long-term outcomes and survivorship following cementless versus cemented fixation during robotic-assisted TKA.

## Introduction

The most common indication for revision surgery following primary total knee arthroplasty (TKA) is aseptic loosening [1], for which young age at the time of index procedure and obesity have been shown to be risk factors [2,3]. With an increasing number of young, obese patients undergoing TKA [4], stable component fixation remains a concern for arthroplasty surgeons. Cemented fixation has long been the gold standard due to its excellent survivorship and clinical outcomes [5-7]. On the other hand, cementless fixation first garnered interest among arthroplasty surgeons in the 1980s, although early prosthetic designs failed because they had poor osteoconductive surfaces and were prone to increased wear and osteolysis [8]. As biomaterials have advanced with the development of highly porous metals and cross-linked polyethylene components, there has been renewed interest in cementless fixation for TKA.

There is currently no consensus for this ongoing debate regarding the optimal method of fixation in TKA; however, there has been growing evidence in favor of cementless fixation. While many studies demonstrate similar clinical outcomes and survivorship between these two fixation methods [5,6,9], cementless TKA has been shown to have improved survivorship in morbidly obese patients [10,11]. The long-term stability of cemented TKAs has also recently been called into question as radiostereometric studies have allowed for in vivo analysis of component fixation [12,13]. Results of these studies demonstrated persistent migration of cemented components over long-term follow up, suggesting superior fixation of cementless implants [12,13]. Proponents of cementless fixation believe that osseointegration creates a more physiologic bond between the implant and the bone while preserving bone stock. 

Proper component alignment is another important factor in ensuring long term implant survival following TKA and may be achieved with robotic assistance. Conventional techniques utilizing extramedullary or intramedullary guides for intraoperative implant alignment are surgeon-dependent and do not always result in ideal component positioning and lower limb alignment [14,15]. Robotic-assisted surgery has been developed to increase the accuracy and precision of bone cuts during TKA. This has been shown to improve component positioning and overall lower limb alignment while facilitating soft tissue balancing [16,17]. The use of robotics in TKA has similarly been associated with improved functional outcomes and quality of life scores compared to conventional TKAs [18,19]. However, no study to date has investigated the role of robotic-assisted surgery on postoperative outcomes following cementless versus cemented TKA.

To address this gap, our study aimed to compare outcomes for robotic-assisted cemented and cementless TKAs. The purpose of this study is to compare patient reported outcomes and complication rates between cementless and cemented TKAs performed with a robotic-assisted technique. We hypothesize that patients undergoing cementless TKA will have similar outcomes without increased complication rates compared to their cemented counterparts.

## Materials and methods

This study was a retrospective review of patients who underwent primary robotic-assisted TKA (Ra-TKA) performed by a single fellowship-trained arthroplasty surgeon at our institution from January 1, 2016 to September 1, 2020. After Institutional Review Board approval, potential subjects were identified using OBERD, a patient-reported outcomes database. All patients 18 years of age or older with completed pre-operative Oxford Knee Scores (OKS) and Short Form Health Survey (SF-12) OBERD data were considered eligible for the study. All included patients underwent a primary Ra-TKA procedure.

Medical records were reviewed for patient demographics, component fixation method, postoperative complications (deep vein thrombosis, infection, arthrofibrosis requiring manipulation, and revision surgery), and comorbidities as mentioned in the Elixhauser comorbidity index.

Surgical procedure

TKAs were performed utilizing the Stryker robotic-assisted MAKO system (Stryker, Kalamazoo, MI). Preoperatively, a CT scan was obtained to generate a bone model and individualized surgical plan. A standard medial parapatellar approach was utilized for exposure. Tracker pins were placed on the tibia and femur to establish anatomy and limb alignment. Prior to performing bone cuts, optimal joint balancing and component alignment were determined by manipulating the joint with the virtual software. The robotic arm was used to perform sequential bone cuts. Trial implants were then inserted and the alignment, range of motion, and ligament balance were evaluated. Either press-fit or standard cemented components were then implanted (Stryker Triathlon CR-knee, Mahwah, NJ). Patellar tracking and final component alignment were evaluated prior to wound closure.

Postoperative protocol

Patients were immediately weight-bearing as tolerated postoperatively and began physical therapy the same day of the procedure. All participants completed a standard protocol with a combination of both in-home and outpatient physical therapy focusing on strengthening and range of motion (ROM) exercises. Patients followed up at four weeks, 10 weeks, one year, and two years for radiographic and clinical evaluation.

Patient-reported outcomes

To assess functional outcomes, OKS and SF-12 scores were obtained by OBERD via emails or telephone preoperatively and at six months, one year, and two years postoperatively as part of standard clinical operations. OKS is a patient-reported outcome and is a validated set of 12 questions used to assess post-operative pain and function after TKA. Each question has a range of 0-4 for a summative score out of 48, with 48 being the best possible score (least symptomatic) and 0 being the worst possible score (most symptomatic). SF-12 is also a patient-reported outcome consisting of 12 questions used as a quality-of-life measure, which includes a physical component summary (PCS) and a mental component summary (MCS). Both the SF-12 PCS and MCS range from 0% (worst level of functioning) to 100% (best level of functioning). The OKS and SF-12 have been shown to be the best-performing knee-specific and generic outcome scores in terms of their measurement properties (e.g., validity, reliability, and responsiveness) [20]. The minimal clinically important difference (MCID) for OKS has been established as 5.0 and 4.3 points for improvements in pain relief and function, respectively [21]. The MCID for SF-12 physical scores is 1.8 while the MCID for SF-12 mental scores is 1.5 [21].

Statistical analysis

A priori power analysis was performed based on published MCID for OKS and SF-12 scores [21]. To detect an MCID, sample sizes of 54 in each cohort would achieve 81% power. Accounting for 20% in loss to follow-up, the total sample size required was 135.

Baseline patient demographics were reported with descriptive statistics. Continuous variables were reported as a mean and standard deviation, while categorical or dichotomous variables were reported as proportions. Univariate analysis was performed with an independent t-test for continuous variables. Chi-squared test was applied for differences in proportions and Fisher’s exact test was used as appropriate. Additionally, paired t-test was performed to observe for any significant differences in patient-reported outcomes between two-time points within groups. A p-value cut-off was set at 0.05 for significance.

## Results

Baseline characteristics

Demographics and baseline characteristics of cementless versus cemented cohorts are depicted in Table 1. Three hundred eighty knees in the cementless cohort and 72 cemented knees were included for analysis. The cementless cohort was significantly younger than the cemented group (65.5 ± 8.5 years and 69.9 ± 8.8 years, respectively; p<0.0001). The mean BMI was significantly higher in the cementless cohort compared to the cemented cohort (32.0 ± 5.9 kg/m^2^ and 30.4 ± 6.2 kg/m^2^, respectively, p=0.0346). The cementless cohort also had significantly lower mean Elixhauser Comorbidity Index Scores than the cemented group (2.4 ± 2.6 and 3.0 ± 1.9, respectively, p=0.041). There were no significant differences in the proportion of females or previous knee surgery between the two cohorts (p=0.1048 and p=0.8303, respectively).

**Table 1:** Baseline patient demographics BMI: body mass index, ECI: Elixhauser comorbidity index *Denotes p-value < 0.05

	Cementless (n=380)	Cemented (n=72)	P-value
Age, years (SD)	65.5 (8.5)	69.9 (8.8)	< .0001
BMI, kg/m^2^ (SD)	32.0 (5.9)	30.4 (6.2)	0.0346*
Female sex, n (%)	198 (52.1)	45 (62.5)	0.1048
Laterality, n (%)			
Left	185 (48.7)	43 (59.7)	0.0859
Right	195 (51.3)	29 (40.3)	
Prior surgery, n (%)	127 (33.4)	25 (34.7)	0.8303
ECI	2.4 (2.6)	3.0 (1.9)	0.0410*

Patient-reported outcomes

There were no significant differences between the two cohorts in terms of SF-12 scores (mental and physical) or OKS preoperatively and at six-month, one-year, or two-year follow-up (Table 2).

**Table 2:** Preoperative and postoperative comparisons of patient-reported outcomes

	Cementless (n=380) Mean ± SD	Cemented (n=72) Mean ± SD	P-value
SF-12 Mental			
Preoperative	53.5 ± 10.9	54.0 ± 10.5	0.715
6-month	55.1 ± 8.2	53.9 ± 8.9	0.429
1-year	54.4 ± 8.2	53.8 ± 10.3	0.677
2-year	54.8 ± 7.9	54.3 ± 8.0	0.726
Oxford Knee Score			
Preoperative	23.6 ± 8.9	25.1 ± 7.2	0.177
6-month	39.5 ± 6.9	39.3 ± 6.2	0.887
1-year	41.3 ± 6.8	41.2 ± 6.7	0.967
2-year	42.5 ± 6.6	42.7 ± 6.2	0.805
SF-12 Physical			
Preoperative	32.8 ± 8.5	32.2 ± 8.8	0.596
6-month	44.6 ± 9.7	44.7 ± 10.1	0.943
1-year	46.8 ± 9.9	46.0 ± 10.5	0.630
2-year	46.3 ± 10.7	45.7 ± 10.1	0.721

Complications and revisions

There were no significant differences in the rates of revision surgery, infection, deep vein thrombosis, or arthrofibrosis requiring manipulation between the two cohorts (Table 3).

**Table 3:** Complication and revision surgery rates

	Cementless (n=380)	Cemented (n=72)	P-value
Revision, n (%)	4 (1.1)	0 (0)	0.713
Infection, n (%)	3 (0.8)	1 (1.4)	0.623
Deep Vein Thrombosis, n (%)	2 (0.5)	0 (0)	0.978
Arthrofibrosis requiring manipulation, n (%)	7 (1.8)	3 (4.2)	0.232

## Discussion

Optimal component fixation remains a concern among arthroplasty surgeons to prevent aseptic loosening, which is the most common indication for revision TKA. Cemented fixation has long been the favored mode of fixation during TKA, although cementless fixation may be able to attain more durable long-term fixation once osseointegration has occurred. No study to date has investigated the role of a robotic-assisted surgical technique on outcomes following cemented versus cementless TKA. The purpose of this study was therefore to compare outcomes between cementless and cemented Ra-TKAs. At short-term follow-up, there were no significant differences in patient-reported outcomes or complication rates.

Cementation is currently the gold standard and most utilized mode of fixation during TKA. The long-term integrity of cemented fixation compared to cementless fixation has recently been questioned due to the poor resistance to shear and tensile forces of cement. Over time, cement can fatigue and develop micro-fractures that cannot be remodeled. Cementless fixation through osseointegration offers a more dynamic method of fixation which can be remodeled over time, potentially leading to more robust long-term fixation. However, early cementless implants were compromised by designs that were not suitable for adequate osseointegration to occur and were frequently complicated by early failure [22-24]. With the advent of improved biomaterials including highly porous metals and cross-linked polyethylene components, more recent studies have shown promising results regarding the quality of fixation attained during cementless TKA. Radiostereometric analysis has been utilized as an in vivo method of quantifying prosthesis migration to provide insight into the durability of component fixation. At an average 12-year follow-up, Laende et al. found the amount of inducible component displacement to be significantly lower among patients who underwent cementless TKA, suggesting superior fixation in this cohort of patients [12]. Clinical trials are warranted to investigate the long-term survivorship of these newer, more advanced cementless implants.

In our study, both cementless and cemented TKAs had improved clinical outcomes compared to their preoperative status without differences in patient-reported outcomes noted between the two cohorts. There were similarly no significant differences in the frequency of complications or revision surgeries at short-term follow-up. Several previous studies have compared these methods of fixation with similar results. Nam et al. reported equivalent clinical outcomes without differences in revision surgery rates between cemented and cementless versions of the same cruciate-retaining implant utilized in our study (Stryker Triathlon CR-knee, Mahwah, NJ) [9]. Kim et al. performed simultaneous bilateral TKAs to compare cemented and cementless fixation in 80 patients using the NexGen cruciate-retaining system (Zimmer Biomet) and similarly found no differences in clinical outcomes or complication rates between the two methods of fixation [6]. Continued surveillance of new surgical techniques and cementless designs is necessary. The results of this study demonstrate similar outcomes between a cementless prosthesis and its cemented counterpart. To our knowledge, this is the first study to date to compare outcomes of cemented and cementless TKAs utilizing a robotic-assisted surgical technique.

With the increase in the utilization of robotic assistance during TKA [25,26], it is important to critically evaluate and maximize its known benefits. Currently, known benefits of Ra-TKA over conventional TKA include: 1) decreased readmissions [25], 2) decreased time to hospital discharge [27], and 3) improved implant positioning [28]. Unfortunately, since our study involved the comparison of implant fixation techniques among TKAs with robotic assistance, evaluating the direct role or effect of robotic assistance on these implant fixation techniques was challenging since this was not the main purpose of our study. However, a previous study of cemented TKAs suggested adjustment of final component positioning compared to TKAs performed using computer navigation [29]. Considering this finding, cemented fixation may have an advantage over cementless fixation in its ability to accommodate and mitigate some errors in positioning. In short, the lack of clinical differences observed between the cementless and cemented TKAs in our study may be explained by the advantage robot assistance confers on increasing precision in cementless fixation. Future research involving robot assistance and implant fixation techniques should directly evaluate the effect of robot assistance on implant positioning.

This study has several limitations. First, this is a single-surgeon study and results may not be generalizable. Second, there are limitations inherent to the retrospective nature of this study. The method of fixation utilized at the time of surgery was not randomized and therefore our results do not indicate that all patients are appropriate candidates for cementless fixation for TKA. Further research is warranted to better understand the ideal patient selection for each method of fixation. Third, the duration of follow-up was short in the context of an arthroplasty procedure, and continued surveillance at the longer duration of follow-up is necessary to ensure that differences in outcomes between fixation methods do not become apparent over time. 

## Conclusions

This retrospective review of patients undergoing Ra-TKA demonstrated equivalent patient-reported outcomes and complication rates of a cementless TKA compared to its cemented counterpart. Further research is warranted to better understand the survivorship of cementless versus cemented TKAs and the role of robotic-assisted surgery during component fixation.
